# Genetic and Hormonal Regulation of Chlorophyll Degradation during Maturation of Seeds with Green Embryos

**DOI:** 10.3390/ijms18091993

**Published:** 2017-09-16

**Authors:** Galina Smolikova, Elena Dolgikh, Maria Vikhnina, Andrej Frolov, Sergei Medvedev

**Affiliations:** 1 Department of Plant Physiology and Biochemistry, St. Petersburg State University, St. Petersburg 199034, Russia; s.medvedev@spbu.ru; 2 All-Russia Institute for Agricultural Microbiology, St. Petersburg State University, St. Petersburg 199034, Russia; dol2helen@yahoo.com; 3 Department of Bioorganic Chemistry, Leibniz Institute of Plant Biochemistry, 06120 Halle (Saale), Germany; vikhnina@gmail.com (M.V.); Andrej.Frolov@ipb-halle.de (A.F.); 4 Department of Biochemistry, St. Petersburg State University, St. Petersburg 199034, Russia

**Keywords:** abscisic acid (ABA), chloroembryo, chlorophyll catabolic enzymes (CCE), chlorophyll degradation, photosynthesis, *Pisum sativum*, residual chlorophylls, seed maturation, seeds, *STAY GREEN* (*SGR*)

## Abstract

The embryos of some angiosperms (usually referred to as chloroembryos) contain chlorophylls during the whole period of embryogenesis. Developing embryos have photochemically active chloroplasts and are able to produce assimilates, further converted in reserve biopolymers, whereas at the late steps of embryogenesis, seeds undergo dehydration, degradation of chlorophylls, transformation of chloroplast in storage plastids, and enter the dormancy period. However, in some seeds, the process of chlorophyll degradation remains incomplete. These residual chlorophylls compromise the quality of seed material in terms of viability, nutritional value, and shelf life, and represent a serious challenge for breeders and farmers. The mechanisms of chlorophyll degradation during seed maturation are still not completely understood, and only during the recent decades the main pathways and corresponding enzymes could be characterized. Among the identified players, the enzymes of pheophorbide *a* oxygenase pathway and the proteins encoded by *STAY GREEN* (*SGR*) genes are the principle ones. On the biochemical level, abscisic acid (ABA) is the main regulator of seed chlorophyll degradation, mediating activity of corresponding catabolic enzymes on the transcriptional level. In general, a deep insight in the mechanisms of chlorophyll degradation is required to develop the approaches for production of chlorophyll-free high quality seeds.

## 1. Introduction

In green plants, photosynthesis is the principle route, supplying developing embryos with assimilates [1,2,3]. Because of this, it is recognized as an important factor affecting plant seed stress tolerance and productivity [4]. Thereby, in many species, photosynthetic reactions are localized not only in leaves, but also in developing green embryos and surrounding tissues of the mother plant, i.e., seed coat and pericarp [1,3,5,6,7,8,9,10]. Thus, organic compounds, required for seed development, at least partly, can be synthesized by photosynthetic membranes of seed tissues. Indeed, chlorophyll synthesis begins already at the stage of globule, increases in course of embryo development, and inhibited at the final steps of seed maturation [11]. In previous decades, chlorophylls were found in embryos of hundreds plant species, including agriculturally important crops [3,6,12,13].

At the late stages of embryogenesis, seeds enter the period of dormancy, which is accompanied by dehydration, disintegration of photosynthetic apparatus, and chlorophyll degradation. Remarkably, the latter process often remains incomplete, and residual chlorophylls can be easily detected in seed tissues of many plant species [14]. The obvious reason for this is disturbance of the constitutive pathways of chlorophyll degradation at the last maturation steps [3]. Most often, this phenomenon is underlied by unfavorable environmental factors, like drought and extremely high or low temperatures [15,16]. Importantly, the presence of residual chlorophylls in seeds dramatically reduces their tolerance to various environmental stresses [14,17,18] and results in significant losses of crop harvest yields due to the so-called “green seed problem” [15,16,19,20]. Therefore, it can be considered as one of the main factors negatively affecting crop productivity and quality of related products. It is especially important for oils, obtained from soybean and oilseed rape seeds [16,21]. Interestingly, the seeds, containing residual chlorophylls, are often treated as immature. However, recently we demonstrated that green pea seeds successfully pass through all steps of embryogenesis and can be considered as physiologically mature [22].

Obviously, the presence of residual chlorophylls might be underlied by disturbance of their catabolism. During the previous decade, six types of enzymes, involved in chlorophyll catabolism (also referred to as chlorophyll catabolic enzymes, CCEs), were identified: chlorophyll *b* reductase, [23,24,25], 7-hydroxymethyl chlorophyll *a* reductase [26], Mg^2+^-dechelatase [27,28], pheophytinase [29], pheophorbide *a* oxygenase [30], and reductase of red chlorophyll catabolite, RCC [31].

Surprisingly, annotation of the gene, responsible for the color of pear cotyledons and originally designated as B and then as I [32], was completed only 140 years after the well-known work of Mendel (1866) was published [33]. Thus, it was found, that the yellow or green color of pea seeds is determined by the family of *STAY-GREEN* (*SGR*) genes, characterized by a high level of conservatism [34,35]. Generally, the products of *SGR* genes act as the key regulators of chlorophyll degradation, destabilizing protein-pigment complexes and increasing availability of chlorophylls for cleavage by chlorophyll catabolic enzymes [25,36,37]. All SGR proteins have a C-terminal cysteine-containing motif in their structure, distinguishing them from the SGR-like ones [38]. Generally, the green color of seeds is underlied by a mutation in the *SGR* genes encoding the SGR proteins involved in the destruction of chlorophyll during seed maturation or onset of leaf senescence [34,35]. Analysis of *sgr* missense mutants revealed several amino acid residues critical for the functional activity of SGR proteins [39,40], although the expression levels of *SGR* genes were not affected.

The general physiological and biochemical role of plant CCEs and SGRs were intensively studied over the recent decade [23,36,41,42]. Although, the mechanisms of their transcriptional regulation are still not completely understood, an essential body of information about possible functions of SGR and CCE proteins in plants can be obtained in leaf senescence experiments [23,36,41,42,43,44]. This approach revealed a possible functional association of SGR proteins with dissociation of chlorophyll-protein complexes in chloroplasts, which makes chlorophylls susceptible to degradation [25,45]. However, the role of the SGRs and CCEs in specific reactions of chlorophyll degradation in seeds with green embryos is still insufficiently characterized, and underlying mechanisms are unclear.

Therefore, in this review we summarize the literature data on chlorophyll degradation in maturing seeds and critically discuss the pathways of genetic and hormonal control of this process with a specific emphasis on *SGR* genes and their products as the key regulators of chlorophyll catabolism.

## 2. Photosynthesis in Seeds with Green Embryos

Based on the presence or absence of chlorophylls in embryos, angiosperms can be divided into the groups of chloroembryophytes and leucoembryophytes [3,12]. In this context, chloroembryophytes are the plants, containing chlorophylls in developing seeds, which photosynthesize during the whole period of embryogenesis. This group is represented by such crops as soybean (*Glycine max* L.), peas (*Pisum sativum* L.), common beans (*Phaseolus vulgaris* L.), broad beans (*Vicia faba* L.), chickpeas (*Cicer arietinum* L.), oilseed rape (*Brassica napus* L.), cabbage (*Brassica oleracea* L.), radish (*Raphanus sativus* L.), mustard (*Brassica nigra* L.), cotton (*Gossypium hirsutum*), and common flax (*Linum usitatissimum*) [12,14,46]. In these plants, the seed chloroplasts are formed already at the stage of globule, and contain chlorophylls *a* and *b*. Thereby, all photosynthetic complexes, i.e., photosystems I and II, their antenna complexes, *b*_6_*f* cytochrome complex and ATP synthase, are present in the stoichiometry required for efficient photosynthesis [3,5,47,48]. As can be evidenced by a high ribulose-1,5-bisphosphate carboxylase/oxygenase (RuBisCO) activity, such seeds fix СО_2_ [1,7,49].

**Figure 1:**
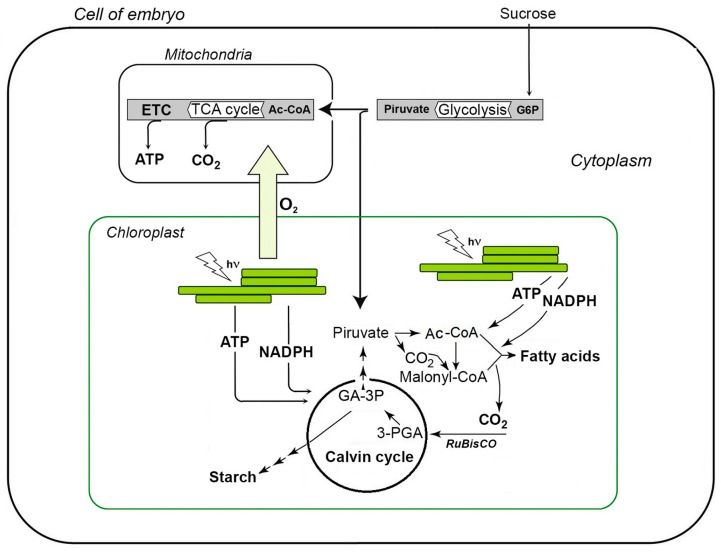
Scheme of carbon and energy metabolism in seeds with green embryo. TCA cycle, cycle of tricarboxylic acids (Krebs cycle); ETC, electron transport chain; G6P, glucose-6-phosphate; Acetyl-CoA, acetyl-coenzyme A; 3-PGA, 3-phosphoglyceric acid; GA-3P, glyceraldehyde-3-phosphate; hν, photon.

Generally, the biochemistry of embryonic photosynthesis is fundamentally different from the reactions occurring in leaf. First, the activity of the seed photosynthetic apparatus results in accumulation of reserve macromolecular compounds, rather than monosaccharides, as it occurs in leaves [1,3,5,7]. Therefore, the main function of seed chloroplasts is the synthesis of NAD(P)H and ATP, required for metabolizing of sucrose to acetyl-coenzyme A (acetyl-CoA), fatty acids, and finally, to triacylglycerides [50,51]. Secondly, the main carbon source for seed photosynthesis is not atmospheric CO_2_, but sucrose, coming from the tissues of the mother plant, and CO_2_, released during seed respiration [1,3,52]. In detail, sucrose is cleaved by invertase in *α*-glucose and *β*-fructose, which are further involved in glycolysis (Figure 1). Resulted pyruvate undergoes decarboxylation, and acetyl-CoA formed is involved in fatty acid biosynthesis [50]. Carbon dioxide, generated in the pyruvate dehydrogenase reaction, is re-assimilated by RuBisCO to form 1,3-diphosphoglyceric acid, the primary product of Calvin cycle [1,3,49]. Most probably, the initial reactions of the Calvin cycle, i.e., СО_2_ fixation and formation of reduced trioses (glyceraldehyde-3-phosphate and dihydroxyacetone phosphate), are sufficient for seed development [53]. Not less important, that oxygen, generated during photosynthesis, prevents hypoxia and supports mitochondrial respiration in developing seeds [5,46,50,54,55].

Thus, the primary roles of the embryonic photosynthesis are (i) accumulation of reserve polymers in developing seeds and (ii) supply them with oxygen. Due to this type of carbon metabolism, seed chloroplasts can be considered as photogeterotrophic organelles. At the late steps of embryogenesis, seeds undergo dehydration and enter dormancy. Thereby, chloroplasts accumulate reserve nutrients and transform in amylo- and elaioplast in parallel to degradation of chlorophylls [8,56,57].

## 3. Catabolism of Chlorophylls in Plants

Due to a world-wide increase in production of plant oils and their esterification products (especially in the context of biofuel industry), the mechanisms of chlorophyll degradation during seed maturation attracts increasing interest in research [58]. Indeed, as chlorophyll and its derivatives are good photosensitizers, and are able to trigger oxidative degradation of fatty acids, contamination of extracted vegetable oils and biodiesel with these compounds can reduce their quality and shelf life [59].

**Figure 2:**
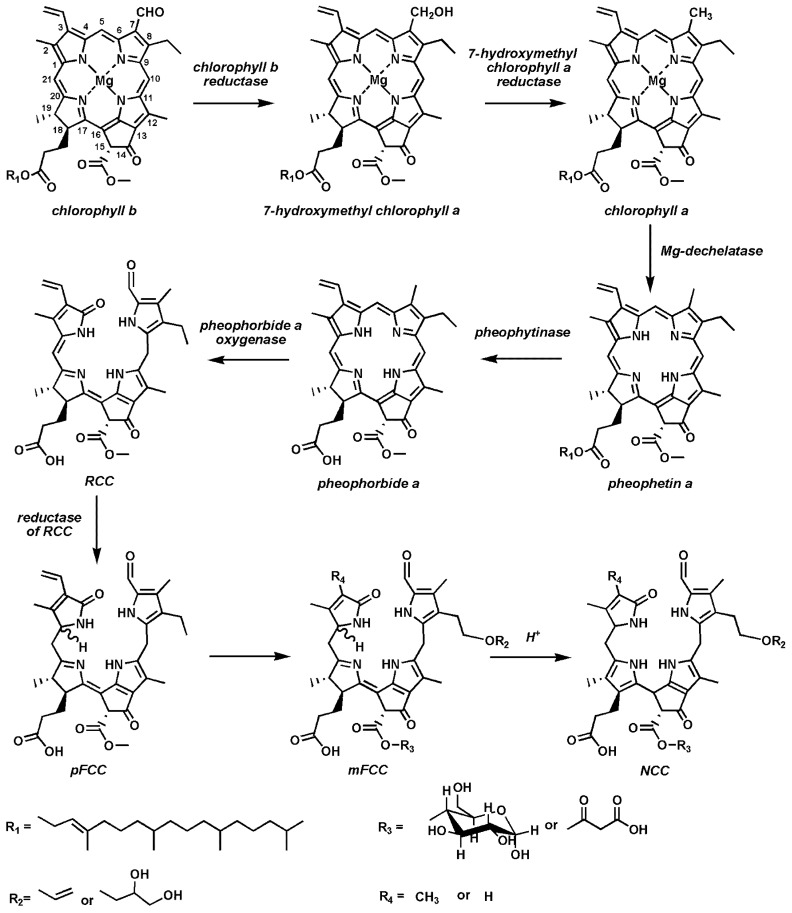
The pathway of chlorophyll degradation in the higher plants. RCC, red chlorophyll catabolite; pFCC, primary fluorescent chlorophyll catabolite; mFCC, modified fluorescent chlorophyll catabolite; NCC, non-fluorescent chlorophyll catabolite.

In general, although seeds receive assimilates from the mother plant, the pathways of seed chlorophyll synthesis and degradation appear to be similar to those in a leaf. In leaves, degradation of chlorophylls mostly relies on so-called the pheophorbide *a* oxygenase (PaO) pathway. After a comprehensive characterization of the corresponding principle chlorophyll catabolic enzymes (CCEs) encoded by chlorophyll catabolic genes (CCGs), it became possible to disclose the main steps of this metabolic pathway (Figure 2). Thus, the catabolism of chlorophylls begins with conversion of chlorophyll *b* to 7-hydroxymethyl chlorophyll *a* [23,24,25]. In the next step, catalyzed by hydroxymethyl chlorophyll *a* reductase, hydroxymethyl chlorophyll *a* is converted to chlorophyll *a* [26]. Then, Mg^2+^-dechelatase removes a magnesium ion from a chlorophyll *a* molecule with formation of pheophetin *a* [27,28,36]. Recently, it was shown that Mg^2+^-dechelatase is encoded by the Mendel’s gene‒*SGR/NYE* (*STAY GREEN/NON-YELLOWING*), responsible for the color of cotyledons [28,60]. It was also demonstrated, that recombinant SGR proteins are able to withdraw Mg^2+^ not only from free chlorophyll molecules, but also from chlorophylls located in the pigment-protein complexes. Thereby, SGR1 and SGR2 proteins showed a relatively high affinity for the magnesium ion of chlorophyll *a* molecule, whereas their dehelatizing potential in respect of chlorophyll *b* and chlorophyllide *a* turned to be rather low [28]. On the other hand, SGR-like (SGRL) protein had a higher efficiency of magnesium withdrawal from a chlorophyllide molecule in comparison to chlorophyll *a*.

The further step, i.e., cleavage of pheophytin *a*, is accompanied with formation of pheophorbide *a* and phytol, and catalyzed by pheophytin pheophorbide hydrolase pheophytinase [29]. This enzyme is highly specific for cleavage of phytol from pheophytin, but not from chlorophyll. Thus, pheophytinase mutants are not able to degrade chlorophyll, and therefore have a stay green phenotype [29]. Degradation of the pheophorbide macrocycle is catalyzed by pheophorbide *a* oxygenase (PaO, Figure 2). This enzyme contains a Rieske type Fe–S cluster, and catalyzes an oxidative cleavage of the double bond between C4 and C5 pheophorbide atoms with formation of an unstable red chlorophyll catabolite (RCC) [30,57,61].

Pheophorbide *a* serves as a substrate for PaO and pheophorbide *b* acts as a competitive inhibitor. In the maturing seeds and senescent leaves of oilseed rape (*Brassica napus*), two genes encoding PaO (*BnPaO_1_* and *BnPaO_2_*) were identified [15]. However, only one of them, namely *BnPaO_2_*, is expressed during maturation of seeds. Remarkably, a low temperature treatment at 0–5 °C leads to disruption of the PaO-dependent chlorophyll degradation pathway and accumulation of so-called “residual” chlorophylls in mature seeds [14,15,19,59,62]. At the terminal stages of the chlorophyll catabolism, RCC is reduced by the C20-C1 double bond of the macrocycle, and an uncolored primary fluorescent chlorophyll catabolite (pFCC, Figure 2) with a strong blue fluorescence is formed. This reduction is catalyzed by red chlorophyll catabolite reductase, a highly soluble stroma protein [31]. Afterwards, pFCC is translocated to cytoplasm, and involved in further transformations at the C3-, C8-, and C13- macrocycle atoms [61,63,64]. Finally, so-called modified fluorescent chlorophyll catabolites (mFCCs) are formed (Figure 2) and translocated into vacuole by ATP-binding cassettes (ABC) transporters. In this compartment, acid-driven migration of double bond from C15 to C16 position of mFCC destroys the conjugated *π*-electron system leading to formation of non-fluorescing chlorophyll catabolites (NCC) [61,64].

To summarize, the main steps and key enzymes of chlorophyll metabolism were described over the previous decade. Thereby, multiple mutants with the stay-green phenotype of maturing seeds and/or senescent leaves could be obtained and comprehensively characterized [34,35,40,65,66,67,68].

## 4. The Role of *STAY-GREEN* Genes in Degradation of Seed Chlorophylls

During the recent decades, the *SGR* genes were described in various plant species, including world-wide cultured crops [34,35,40,65,69,70]. In the most easy and straightforward way, the functions of these genes can be addressed by corresponding *stay-green* (*sgr*) mutants [71].

Currently, based on the presence or absence of the photosynthetic activity and kinetics of leaf senescence, five types of *stay-green* (*sgr*) mutants are distinguished (Table 1). According to Thomas and Howarth (2000), these five types can be divided in two principal categories, usually termed functional and cosmetic mutants, in which leaf senescence onset occurs without and with a loss of photosynthetic activity, respectively [72]. The functional mutants are represented with the types A and B, whereas the type C’s are referred to as cosmetic mutants [72]. Thereby, the mutants of type B are characterized with lower rates of senescence onset, in comparison to those of the type A. As in both mutant types duration of the photosynthetically active developmental stage is prolonged in comparison to the wild type, they are defined as the functional ones. The chlorophylls of the type C mutants do not degrade with the senescence onset, while photosynthesis and senescence-related events proceed at the rates, comparable to the wild type [67,72]. For example, the famous green cotyledon pea mutant, reported by Gregor Mendel (1866), belongs to the C-type [66]. The mutants of the type D are often referred to as pseudo-*stay-green*, because of drought- or cold-induced leaf death, developing before or during senescence onset [67,72]. Finally, the mutants of type E accumulate extremely high levels of leaf chlorophylls, without an appropriate simultaneous increase in photosynthetic competence [67,72], that results in longer times, required for the loss of green color.

As mentioned above, the green color of pea seeds is determined by a mutation in the *SGR* genes, which encodes proteins responsible for the degradation of chlorophylls in chloroplasts during seed maturation or leaf senescence [34]. In turn, the yellow color of pea seeds is determined by carotenoids, which become visible after completion of chlorophyll degradation.

During the recent decade, homologues of the *SGR* genes were cloned in rice *Oryza sativa* (*OsSGR*), arabidopsis *Arabidopsis thaliana* (*AtSGR1*, *AtSGR2*), pepper *Capsicum annuum* (*CaSGR*), tomato *Solanum lycopersicum* (*SlSGR*), barley *Hordeum vulgare* (*HvSGR*), and soybean *Glycine max* (*GmSGR1*, *GmSGR2*) [40,64,68,73,74]. In the case of the presence of several *SGR* genes in the plant genome, they might show a high degree of similarity (e.g., 91% between the genes *GmSGR1* and *GmSGR2* in soybean) and functional equivalence [16]. The sequence analysis of *SGR* genes in rice allowed characterization of a new gene family encoding the proteins localized in chloroplasts and involved in degradation of photosynthetic complexes of photosynthetic membranes [68]. The levels of *SGR* expression are directly related to the degradation of both chlorophyll-binding proteins and chlorophylls during the maturation of seeds and the onset of leaf senescence [68]. In this context, the stability of chlorophyll-binding proteins, and, in particular, of the light-harvesting complex II subunits, during seed maturation and leaf senescence is the common feature of all *sgr* mutants [68]. Generally, the levels of *SGR* gene expression increase in course of seed maturation [75]. Therefore, the plants with different contents of residual chlorophylls can be distinguished. For example, both *GmSGR1* and *GmSGR2* genes demonstrate similar expression patterns during maturation of soybean seeds. Thereby, expression of the both genes correlated with the degradation of chlorophylls [16].

Although the *SGR* genes were cloned several years ago, their role in the regulation of chlorophyll degradation is still poorly understood. Nevertheless, based on the sequence features of the SGR proteins, the consensus motifs, related to their functional activity, can be assigned. Thus, such polypeptides contain a conservative core and a cysteine-rich C-terminal Cys-X_3_-Cys-X-Cys-Cys-Phe-Pro-X_7_-Pro motif [38]. The role of this motif is still unknown, although its cysteine residues might be involved in formation of intra- and intermolecular disulfide bonds or could act as redox sensors [66]. Interestingly, this apparently functionally important C-terminal motif is absent in the sequences of SGR-like proteins, e.g., the products of Arabidopsis *AtSGR3* and rice *OsSGR2* or *OsSGR3* genes [39,73]. Functional analysis of missense and insertion *sgr* mutants revealed specific amino acid residues in the sequences of SGR proteins as critically important for their activity [39,40]. Corresponding mutations were described in *sgr* locus of rice *O. sativa* and *cl* locus of pepper *C. annuum* [39,40].

Although *sgr* missens mutants were characterized with relatively high levels of residual *SGR* gene expression, the functional activity of SGR proteins can be essentially affected by substitution of specific amino acid residues [39,40]. Thus, transient expression of full-sized pea or rice SGR proteins in *Nicotiana benthamiana* leaves, after infiltration of an appropriate agrobacterial construct, led to accelerated leaf senescence [38,68]. However, infiltration of mutant constructs did not result in such effects. Further immunoprecipitation experiments with *SGR* products demonstrated a specificity of their binding with proteins of the light harvesting complex II (LHCP) [68]. Transient overexpression of *SGR* in *Nicotiana benthamiana* and an in vivo pull-down assay show that *SGR* interacts with LHCPII, assuming formation of the SGR-LHCPII complex in the thylakoid membranes. This might indicate the possible role of SGR in destabilization of pigment-protein complexes, which leads to their degradation and makes chlorophyll susceptible to degradation by catabolic enzymes [28,37,68].

## 5. Role of Abscisic Acid (ABA) in Degradation of Chlorophylls and Seed Maturation

Generally, abscisic acid (ABA) is involved in regulation of the key processes accompanying seed maturation, i.e., synthesis and transport of carbohydrates, accumulation of reserve nutrients, tissue dehydration, chlorophyll degradation, and onset of dormancy [76,77,78,79,80]. Generally, in the context of ABA tissue contents, maturation of seeds can be considered as a two-step process [81]. During the first step, typically characterized by intensive cell division, accompanying the formation of the embryo and endosperm, the ABA tissue contents are relatively low. After the completion of embryo formation, the cellular growth switches to the elongation mechanism due to an ABA-mediated inhibition of cell division and arrest of the cell cycle in the G1–S phase. This event is accompanied with a steady increase of ABA tissue contents, underlying the changes in the transport of monosaccharides and amino acids, which results in accumulation of reserve biopolymers. The observed increase in ABA contents is underlied by the intake of this phytohormone from the mother plant, as well as in situ synthesis in the seed embryo and endosperm [80,81,82].

Normally, degradation of chlorophylls is a part of the whole seed maturation process. The role of ABA in this process was comprehensively addressed in multiple studies [20,83]. Thus, it was shown, that a treatment with ABA at the air humidity of 86% enhances degradation of chlorophylls in green seeds of oilseed rape [83]. Thereby, the rates of seed chlorophyll degradation are higher, than the rates of seed dehydration [20]. A gradual decrease of air temperature to −5 °C during 3 h results in essential enhancement of seed dehydration [20]. When the seed water contents drops below 35–45%, degradation of chlorophylls is suppressed, whereas the levels of ABA dramatically increase [20]. As degradation of seed chlorophylls is controlled by the *SGR1* gene [34], the mechanisms of its transcriptional regulation and the role of ABA in this process need to be addressed. In this context, the ABA-dependent transcription factor ABI3 (abscisic acid insensitive 3) seems to be the most promising candidate for the role of the SGR activity regulator [84]. Indeed, this protein is involved in control of the final steps of seed maturation, i.e., dehydration, chlorophyll degradation and onset of dormancy [81,85], and represents, therefore, a promising target for genomic intervention. Accordingly, the first ABA-insensitive Arabidopsis mutant *abi3* (*abscisic acid ins3*) was characterized by Ooms et al. already in 1993 [86]. The seeds of this mutant could not enter dormancy, were not resistant to dehydration, and their chlorophylls were not involved in degradation. Later, Parcy et al. (1997) demonstrated seed localization of *ABI3* expression [77]. One decade later, Clerkx et al. (2003) showed that *ABI3* is involved in the regulation of several features, critical for seed viability: onset of a dormancy period, degradation of chlorophylls, and ability to survive long storage periods [87].

The most solid evidence for the key role of *ABI3* in the regulation of chlorophyll degradation during seed maturation was recently obtained by Delmas et al. (2013) [84]. In experiments with the *A. thaliana* mutant *abi3-6*, earlier obtained by Nambara et al. (1994), the authors found two independent embryogenesis programs to be under the control of ABI3 [76]. According to the authors, this transcription factor was involved in (i) development of seed tolerance to dehydration and (ii) degradation of chlorophylls. In the latter process, it was shown to act as the transcription regulator of *SGR* genes [84]. As the *abi3-6* stay green phenotype was not observed in leaves of the experimental plants, the effect of ABI3 on the degradation of seed chlorophylls was attributed as seed-specific. Moreover, it was demonstrated recently, that ABI3 functions as the master regulator of degreening through transcriptional control of *SGR1* and *SGR2* [84]. However, the ABA-related signaling cascade, controlling the degradation of embryonic chlorophylls during seed maturation, is much more complex and includes a plenty of other proteins besides SGR1 and SGR2 [88,89].

As already mentioned, in the first step of chlorophyll degradation, catalyzed by chlorophyll *b* reductase, chlorophyll *b* is converted to 7-hydroxymethyl chlorophyll *a* [23,90,91]. In rice and Arabidopsis, this enzyme is represented by two isoforms—NYC1 (NON-YELLOW COLORING 1) and NOL (NYC1-Like) [24,25]. The senescent leaves and mature seeds of *nyc1/nol* mutants have a stay green phenotype, which is accompanied with up to ten-fold increase of chlorophyll contents in comparison to the seeds of wild type plants [92]. Generally, a characteristic feature of the most ABA-regulated genes is the nucleotide sequence PyACGTGGC localized in the gene promoter region and usually referred to as ABA-response element (ABRE) [81]. Sequencing of the *NYC1* gene promoter revealed a potential ABA-response element with the sequence CACGTGTC [92]. Further analysis of the changes in electrophoretic mobility of DNA-ABA complexes (addressed by a electrophoretic mobility shift assay, EMSA) revealed binding of the ABA-dependent transcription factor ABF4 to ABRE localized in the *NYC1* gene promoter. This interaction indicates that expression of the *NYC1* gene might be controlled by ABA. To confirm this assumption, the authors compared the levels of *NYC1* expression in embryos of the mutant line *abi3* and the wild type. The analysis revealed a suppression of the *NYC1* expression in developing Arabidopsis seeds due to inactivation of related regulatory genes (e.g., *abi3*). Thus, degradation of chlorophylls in maturing seeds is, at least partially, controlled by ABA via the control of *NYC1* expression.

It is important to note, that ABA plays an important role in regulation of chlorophyll degradation not only in the period of seed maturation, but also during onset of leaf senescence. However, the ABA-dependent mechanisms of gene expression control of chlorophyll degradation are apparently different in seeds and leaves. Recently, Sakuraba et al. (2016) addressed the expression patterns of the ABA-dependent transcription factors [89]. The analysis revealed much higher levels of ABI3, ABI4, SGR1, and NYC1 expression in maturing seeds in comparison to senescent leaves. In conclusion, other genes, besides *SGR* and *NYC1*, are controlled by ABA. For example, the products of such genes are pheophytinase (PPH, pheophytin pheophorbide hydrolase) and pheophorbide *a* oxygenase (PaO) [93,94].

## 6. Residual Chlorophylls in Mature Seeds: The Problem of ”Green Seeds”

At the last step of embryogenesis, accumulation of reserve biopolymers is accompanied with disintegration of grana, inhibition of photosynthesis, and degradation of seed chlorophylls [22]. Most probably, all these events are triggered by dehydration of the seed, and disruption of sucrose flux from the mother plant to the developing embryo [95]. Although the degradation of chlorophylls in maturing seeds and loss of their green color are expected to be complete under field conditions, in practice, it is not always the case [14]. Thus, breeders often report contamination of planting material with green seeds containing high quantities of non-destroyed residual chlorophylls [16,21]. This phenomenon is strongly undesired, as residual chlorophylls dramatically reduce viability of seeds and shelf life of crops and related products [14,17,18,87,96].

This so-called problem of “green seeds” becomes an important factor affecting crop yields, especially in production of oilseed rape and soybeans. Indeed, even a short-term sub-lethal freezing initiates a strong dehydration of pods and seeds, which leads to a partial or complete inhibition of the enzymes involved in the degradation of seed chlorophylls [15,19]. Therefore, spring frosts result in a dramatic increase of chlorophyll contents in the seeds of oilseed rape, and essential economic losses for farmers [97]. Indeed, the presence of more than 6% of green seeds in the material used for the pressing of vegetable oils, results in a dramatic decrease in the quality of obtained products [98]. This deleterious effect of residual chlorophylls is typically manifested with unpleasant taste and smell, as well as shorter shelf life [98].

Not less important is the phenomenon of “green seeds” in producing of soybeans [16]. In this case, the main environmental factors, affecting degradation of seed chlorophylls, are drought and extremely high temperatures, accompanying dramatic climate changes of the last decades and restricting agriculture in some regions [57]. Thus, the drop of seed water contents below 20% results in a dramatic decrease of specific enzymatic activities and efficiency of respiration. Teixeira et al. (2016) have shown that heat and drought stress during seed maturation resulted in impaired expression of *SGR* and *NYC1*, incomplete chlorophyll degradation and *stay-green* phenotype of soybean seeds [16]. Thereby, the rates of chlorophyll degradation essentially decrease. This is true, for example, for the plants grown in Brazilian savanna, where about 45% of world soybean production is localized on the area of 14.5 million hectares [16]. In such areas, even a transient drought results in an increase of residual chlorophyll contents in soybean seeds and decrease of their nutritional value, as well as quality of produced oils [16].

An increasing interest to the problem of “green seeds” can be explained by an explosive growth of a biofuel production during the last decades [58]. Indeed, from the chemical point of view, chlorophylls are effective photosensitizes, i.e., the electrons delocalized in their porphyrin polycycle structure can be easily transferred to excited state in presence of even small amounts of light quanta [3]. Such intermediates can easily initiate oxidative processes in extracted oils and compromise, thereby, their quality [59].

Remarkably, besides environmental stress, the heterogeneity of the seeds, manifested by the differences in their shape, size, mass, seed coat structure and dormancy type, might result in inhibition of chlorophyll degradation during seed maturation [99,100]. This heterogeneity can be explained by the differences in seed location on the mother plant, fruit placenta or compound fruit, and is necessary for adaptation to variable environmental conditions. It can not be excluded, that heterogeneity can be also expressed by a differential presence of residual chlorophylls in seeds. For example, in our previous studies was shown, that chlorophyll contents in the oilseed rape seeds harvested from shoots of the first and second order were 2–2.5-fold higher in comparison to those, located at the main shoot [14]. The seeds of cabbage demonstrated even a higher heterogeneity. Thus, in the phase of wax ripeness, the chlorophyll contents in the seeds of the upper layer were 13-fold higher in comparison to those, located in lower layers [14].

It is important to note, that residual chlorophylls present in physiologically mature seeds and not affecting their germination under optimal environmental conditions, reduce the tolerance of seeds to environmental stress [14,17,18,101]. Therefore, the standard quality assessment protocols established by breeders often include fluorescence analysis of seed chlorophyll contents [17,18,62]. On the physiological level, reduced stress tolerance of chlorophyll-containing seeds is associated with membrane damage and compromised integrity of seed coat, to some extent, associated with insufficient activity of plant antioxidant systems, i.e., the oxidative stress, developing in green seeds, overwhelms defense capacities of the plant organism [3]. Indeed, due to the degradation of thylakoid membranes at the late maturation steps, no photochemical quenching (i.e., conversion of the energy of vertical transitions in the energy of chemical bonds via the synthesis of ATP and NADPH) occurs in such seeds [80]. Hence, in green seeds, the excited electrons can readily reduce molecular oxygen with formation of reactive oxygen species (ROS), which are immediately involved in free radical-mediated oxidative reactions and damage of lipids and fatty acids [3].

## 7. Conclusions

Degradation of chlorophylls not only accompanies the onset of plant leaf senescence, but also can be observed during maturation of seeds with green embryos. However, in some cases, due to environmental stress or the phenomenon of seed heterogeneity, this process remains incomplete, and residual chlorophylls are present in mature seeds. It ultimately results in loss of seed quality, and represents an essential challenge for modern agriculture. Therefore, understanding of the processes behind disturbance of seed chlorophyll degradation is important for sustaining plant productivity and crop quality. Although multiple genes, involved in this process are characterized, the precise mechanisms of chlorophyll degradation during seed maturation are still not completely understood. In this context, several important aspects need to be addressed in the nearest future. First, a deeper insight in genetic aspects of seed color determination is necessary, and search for new genes that are involved seems to be a fruitful approach. This search can rely, for example, on a comparative unbiased analysis of gene and protein expression in seeds, different in maturation rates, color, and quality. The identified gene candidates, characterized by differential expression in various seed groups, can be validated by experiments with appropriate transgenic plants or/and knockout mutants. Most probably, such targets will include the genes, encoding proteins involved in catalysis and regulation of chlorophyll degradation. Finally, the effect of the environmental factors, like temperature, light, and water stress, affecting expression of annotated genes, needs to be addressed.

## Figures and Tables

**Table 1:** Classification of *sgr* mutants based on their phenotypic manifestation.

Types of Mutants	Phenotypic Manifestation of Mutations
A (functional *stay-green*)	Chlorophylls are not degraded, leaf senescence onset is strongly delayed, duration of photosynthetically active stage is prolonged
B (functional *stay-green*)	Chlorophylls are not degraded, leaf senescence onset is slowed down, duration of photosynthetically active stage is prolonged
C (cosmetic *stay-green*)	Chlorophylls are not degraded, but photosynthetic activity and leaf senescence itself remain unaffected
D (pseudo *stay-green*)	Leaves are involved in programmed cell death during or before senescence onset and chlorophyll degradation
E (super green *hyper-green*)	Leaf senescence rates and photosynthetic activity are unaffected, but chlorophylls are strongly up-regulated

The classification of mutants relies on Thomas & Howarth [72] and Kusaba et al. [67].

## Abbreviations

ABA	abscisic acid
ABI	abscisic acid insensitive
CCE	chlorophyll catabolic enzymes
NCC	non-fluorescent chlorophyll catabolite
NOL	NYC1-like
*NYE*	*NON-YELLOWING*
NYC	NON-YELLOW COLORING
mFCC	modified fluorescent chlorophyll catabolite
PaO	pheophorbide *a* oxygenase
pFCC	primary fluorescent chlorophyll catabolite
PPH	pheophytin pheophorbide hydrolase
RCC	red chlorophyll catabolite
*SGR*	*STAY GREEN*
